# Accuracy of digitally fabricated partial coverage ceramic restorations using conventional impression and intraoral scanners

**DOI:** 10.1186/s12903-026-08847-w

**Published:** 2026-06-17

**Authors:** Ahmed Adly, Mohamed Ellayeh, Shaimaa Ahmed Abo El-Farag

**Affiliations:** 1 Fixed Prosthodontics Department, Faculty of Dentistry, Mansoura University, Mansoura, Aldakhlia Egypt; 2 Fixed Prosthodontics Department, Faculty of Dentistry, Mansoura National University, Mansoura, Egypt

**Keywords:** Physical impression, Intraoral scanners, Marginal adaptation, Zirconia, Partial coverage restorations

## Abstract

**Background:**

The purpose of this in-vitro study was to evaluate the accuracy of digitally fabricated partial coverage ceramic restorations with two designs using conventional impression and intraoral scanners.

**Methods:**

Forty-eight sound human maxillary first premolars with homogenous dimensions were partitioned into two equal groups (n_=_24) based on the overlay preparation design; Group (1): teeth prepared with thin conventional design (D1), Group (2): teeth prepared with MODFL (mesial-occlusal-distal-facial-lingual) design (D2). Based on the method of taking impression, each group was subdivided into 3 subgroups (n_=_8); Subgroup (C): conventional impression using polyvinyl siloxane, Subgroup (SC1): scanner type 1 TRIOS 5 (3Shape) and Subgroup (SC2): scanner type 2 Medit i900 (Medit). All fabricated restorations were cemented into corresponding teeth utilizing self-adhesive resin cement (Multilink Speed). All specimens were thermo-cycled for 5000 cycles in a water bath between 5 °C and 55 °C after that marginal adaptation test was done using scan electron microscope. The threshold for statistical significance was established at *p* ≤ 0.05.

**Results:**

SC1 demonstrated the lowest mean marginal gap in D1and D2. The Tukey post-hoc multiple comparison test indicated that statistically significant changes in marginal adaption were mostly related to restorations produced using the SC1, especially in D2. Design 2 overlays generated with SC1(D2SC1) exhibited markedly reduced marginal gap values in comparison to those produced by the conventional impression technique (D2C) (*p* = 0.017) and in relation to D1SC2 (*p* = 0.007), as well as D1 created using the conventional impression method (D1C) (*p* = 0.034). Conversely, the majority of pairwise comparisons between conventional impressions(C) and SC2, irrespective of design type, were not statistically significant.

**Conclusions:**

Digital impressions improved marginal integrity of overlay restorations and offered a clinically effective alternative to conventional silicone impressions within a digital workflow, the TRIOS 5 (3Shape) scanner demonstrated improved marginal adaptation across both preparation designs compared to the other techniques. This may be attributed to its high trueness and precision, as well as its confocal microscopy-based image acquisition technology, with the effect becoming more evident in complex designs.

## Background

The indications for partial coverage restorations (PCRs) have broadened owing to recent advancements in adhesive dentistry and an increased need for aesthetics. A PCR is a form of fixed restoration that does not entirely cover the outer surface of the tooth, including inlays, onlays, laminate veneers, and endocrowns. This careful indirect restoration preserves the integrity of the tooth while maintaining the remaining tooth structure intact [1].

Dental professionals frequently utilize tooth preparation principles to restore damaged dental tissues and improve occlusal force resistance through the application of a full-coverage crown. Nonetheless, these criteria necessitate the reduction of the entire coronal surface, which may be excessively invasive for teeth with intact structural tissues. Consequently, partial coverage restorations, including ceramic or composite overlays, may be regarded as a more conservative treatment alternative. The occlusal surface and functional cusps are primarily impacted in tooth degradation, which influences aesthetics, occlusal vertical dimension, and occlusal stability. To ensure the durability of teeth and restorations, it is essential to preserve the entire dental structure [2].

Consequently, for the restoration of vital teeth with insufficient residual coronal dentin, crown restoration is deemed an unconservative treatment approach. Instead, it is recommended to implement overlay restoration with an altered occlusal scheme to protect the supporting units and the restoration from overload. It is highly recommended minimizing the lateral forces to reduce the risk of fracture. An overlay, or partial crown, is an indirect aesthetic intervention for significant posterior teeth, characterized by a MOD inlay that covers the entire occlusal surface, so protecting all cusps [3].

Various restorative materials have been employed for the fabrication of partial coverage restorations, including cast metal, all-ceramic, and resin-based composites. In the last ten years, the use of cast metal restorations has diminished, primarily due to increasing aesthetic preferences and the rising expense of precious metals. Consequently, tooth-colored substitutes have gained increased popularity [4].

Ceramic restorations combine superior biocompatibility with favorable optical and material characteristics, meeting the expectations of both patients and clinicians. They are intended for veneers, inlays, onlays, crowns, and fixed partial dentures (FPDs). The longevity of ceramic restorations mostly relies on the quality of adhesion and the proper application technique [5].

In fixed prosthodontics, the precision of the impression method and the definitive casts is crucial for the accurate fabrication of restorations, hence avoidance subsequent biological and prosthetic difficulties. Inconsistencies between the intraoral state and the definitive cast may result in restorative misfit and jeopardize the treatment success. The gold standard impression process involves the traditional physical impression utilizing elastomeric materials and stock trays. Despite their low wear resistance and limited setting expansion, stone casts derived from elastomeric impression materials are regarded as a standard for precise fixed dental prostheses. Nevertheless, the traditional process for producing gypsum casts is both time-consuming and labour-intensive. Currently, Computer-aided design and computer-aided manufacturing (CAD-CAM) systems can substitute gypsum casts with printed resin models utilizing intraoral scanners (IOSs) and additive manufacturing techniques [6].

Siqueira et al. [7] stated that IOS technology enhances patient experience, as assessed by overall preference and comfort, and yields dependable prosthodontic outcomes. Furthermore, the digital impressions are time-efficient, facilitating a reduction in working times and consequently expenses when contrasted with conventional impressions. Recent technological improvements in IOS have led to the introduction of devices capable of capturing a full-arch scan in under three minutes.

The advantages of employing digital intraoral scanners over traditional impressions encompass superior accuracy, less chair time, heightened patient comfort, improved communication, greater flexibility, and enhanced visualization. Various models of intraoral scanners offer diverse possibilities, including scanning speed, scanning flow, scanner dimensions, varying user-friendliness, and a broad spectrum of pricing [8].

The durability of permanent restorations depends on the quality of marginal adaptation to the teeth. Marginal areas may create an ideal environment for biofilm accumulation, potentially leading to the development of periodontal disease and dental cavities. The absence of adaptation is strongly linked to gingival irritation, secondary caries, and prosthesis failure; thus, an optimal marginal fit is essential to maintain minimal cement film thickness [9].

In the present study, TRIOS 5 (3Shape) and Medit i900 (Medit) were selected for comparison, as they are different in technology as TRIOS 5 (3Shape) is based on advanced confocal microscopy combined with ultra-fast optical scanning and intelligent image processing. It captures high-resolution 3D data through continuous image acquisition. It focuses on automation and AI-assisted scanning to reduce operator dependency and simplify workflow complexity, but Medit i900 (Medit) utilizes triangulation-based structured light scanning technology, combined with high-speed optical image acquisition and continuous video capture to generate detailed three-dimensional digital impressions [10–12]. The null hypothesis of this in-vitro study assumes that neither the design of preparation nor the method of taking the impression has any effect on marginal adaptation.

## Methods

### Ethical approval

This study adhered to all protocols established by the Local Research Ethics Committee of the Faculty of Dentistry at Mansoura University and obtained clearance number A01011024FP.

### Sample size calculation

Based on Metiner et al. [13] sample size was calculated by the G power program V3.1.9.7Based on effect size of 1.13 using 2-tailed test, $\:\propto\:\:$error = 0.05 and power = = 90%, the sample size in our study was 48 in total, which was allocated into 2 main groups( *n* = 24) then every group will be divided into 3 subgroups (8 in each subgroup).

### Teeth selection

A total of forty-eight whole human maxillary first premolars with well formed roots, exhibiting uniform size and morphology, were selected. All teeth were recently extracted for periodontal or orthodontic reasons and were collected with patient permission at the Oral and Maxillofacial Surgery Department, Faculty of Dentistry, Mansoura University. A digital calliper was employed to ascertain the dimensions of each tooth. The chosen teeth were delineated 2 mm from the cemento-enamel junction [9].

### Teeth cleaning, disinfection and storage

An ultrasonic scaler was employed to debride and cleanse the teeth of surface stains, calculus, and adhering soft tissues. Subsequent low-speed polishing was conducted using polishing paste. In compliance with the directives established by the Centers for Disease Control and Prevention (CDC, 1993), all teeth underwent disinfection for a period of 7 days. Utilizing a 1:10 dilution of 5.25% sodium hypochlorite bleach (Clorox Bleach, Clorox Co., Cairo, Egypt) [14]. To prevent dehydration, teeth were preserved in 0.9% saline at room temperature for the entire testing duration.

### Teeth mounting

To ensure the proper handling of the chosen teeth during following procedures, such as preparation, impression recording, and overlay cementation, their roots were vertically embedded in epoxy resin blocks along their longitudinal axes. Teeth were secured within a cylindrical plastic ring filled with self-curing epoxy resin (KEMAPOXY 150, CMB International, Egypt) using a one-arm dental laboratory parallelometer (Delineador B2, Bio-Art Co., SP, Brazil).

**Fig. 1:**
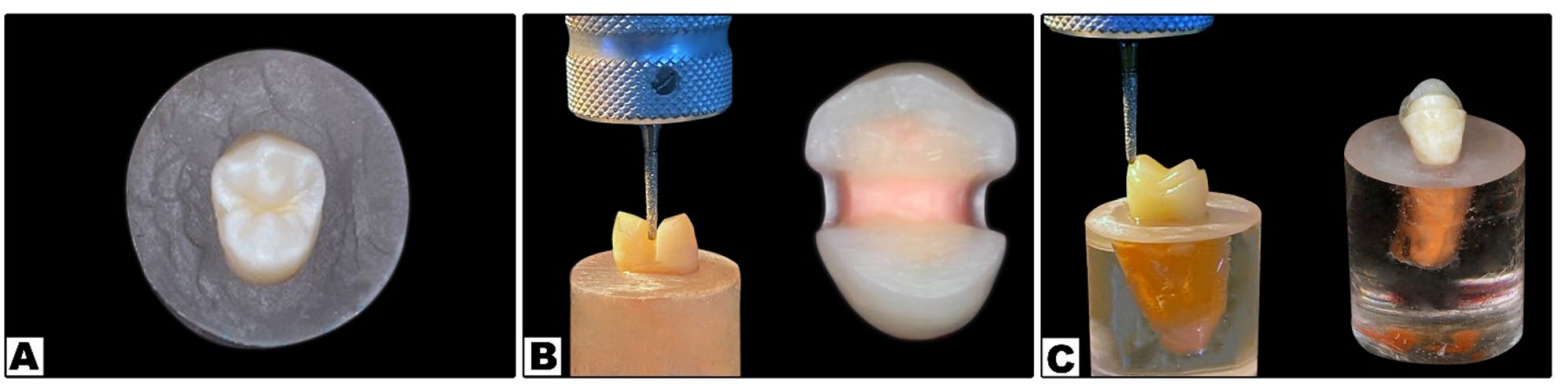
Step-by-step experimental workflow with representative illustrative figures. The procedures included: **A** Teeth mounting, **B** Teeth preparation for overlay design 1 and **C** Teeth preparation for overlay design 2

The simulated alveolar bone level was set 2 mm below the cementoenamel junction (CEJ) level [9]. periodontal ligaments were replicated around the roots following Transitional Wax Technique employing a light-body vinyl polysiloxane (VPS) impression material (Perfit Light-body, Huge Dent, China). All procedures were performed by the same operator [15] (Fig. 1A).

### Samples grouping

The resulted 48 epoxy resin blocks with their mounted teeth were numbered and divided into two equal groups (n_=_24) based on the overlay preparation design for each tooth; Group (1): teeth preparaed with thin conventional design, Group (2): teeth preparaed with MODFL (mesial-occlusal-distal-facial -lingual ) design, Each group was subdivided into three subgroups (n_=_8) based on the method of taking the impression ; Subgroup (C) conventional impression using polyvinyl siloxane, Subgroup (SC1): scanner type1TRIOS 5 (3Shape) and Subgroup (SC2): scanner type 2 Medit i900 (Medit).

### Teeth preparation

Before beginning tooth preparation, a silicon index was made to evaluate the amount of reduction in each tooth. The index was then split in a buccolingual direction into two halves to control tooth structure removal during preparation and the amount of preparation was verified by the preparation putty index and calibrated periodontal probe.

Design 1 (thin overlay): Occlusal surface preparation was started with occlusal guiding grooves by tapered diamond stone using a high-speed headpiece (NSK-Nakanishi International, Japan). The occlusal reduction was done according to occlusal anatomy. 1 mm reduction of palatal cusp and 1 mm reduction of buccal cusp [16, 17]. Axially, a standardized overlay preparation for the selected natural teeth was performed using a dental surveyor (Milling unit BF 2, Bredent GmbH & Co., Germany) and Cavity preparations were executed with an occlusal cavity width maintained at one-third of the intercuspal distance, the pulpal floor depth was 1.5 mm at the central groove region, and each wall exhibited a taper of 3°. The gingival seat in the proximal box was positioned 1 mm above the cementoenamel junction, with a mesiodistal width of 1 mm [2, 18] (Fig. 1B ).

Design 2: mesial occlusal distal facial lingual overlay preparation (MODFL overlay preparation): A 1 mm depth cut was executed to facilitate uniform removal of tooth structure in accordance with the occlusal anatomy. Axially, a standardized overlay preparation was performed using a dental surveyor (Milling unit BF 2, Bredent GmbH & Co., Germany), A broad 1 mm rounded shoulder margin was established with an axial height of 1 mm sloping towards the occlusal surface. All interior preparation angles were rounded, and all surfaces were polished [19] (Fig. 1C).

### Fabrication of overlay restorations

#### Conventional impression technique

**Fig. 2:**
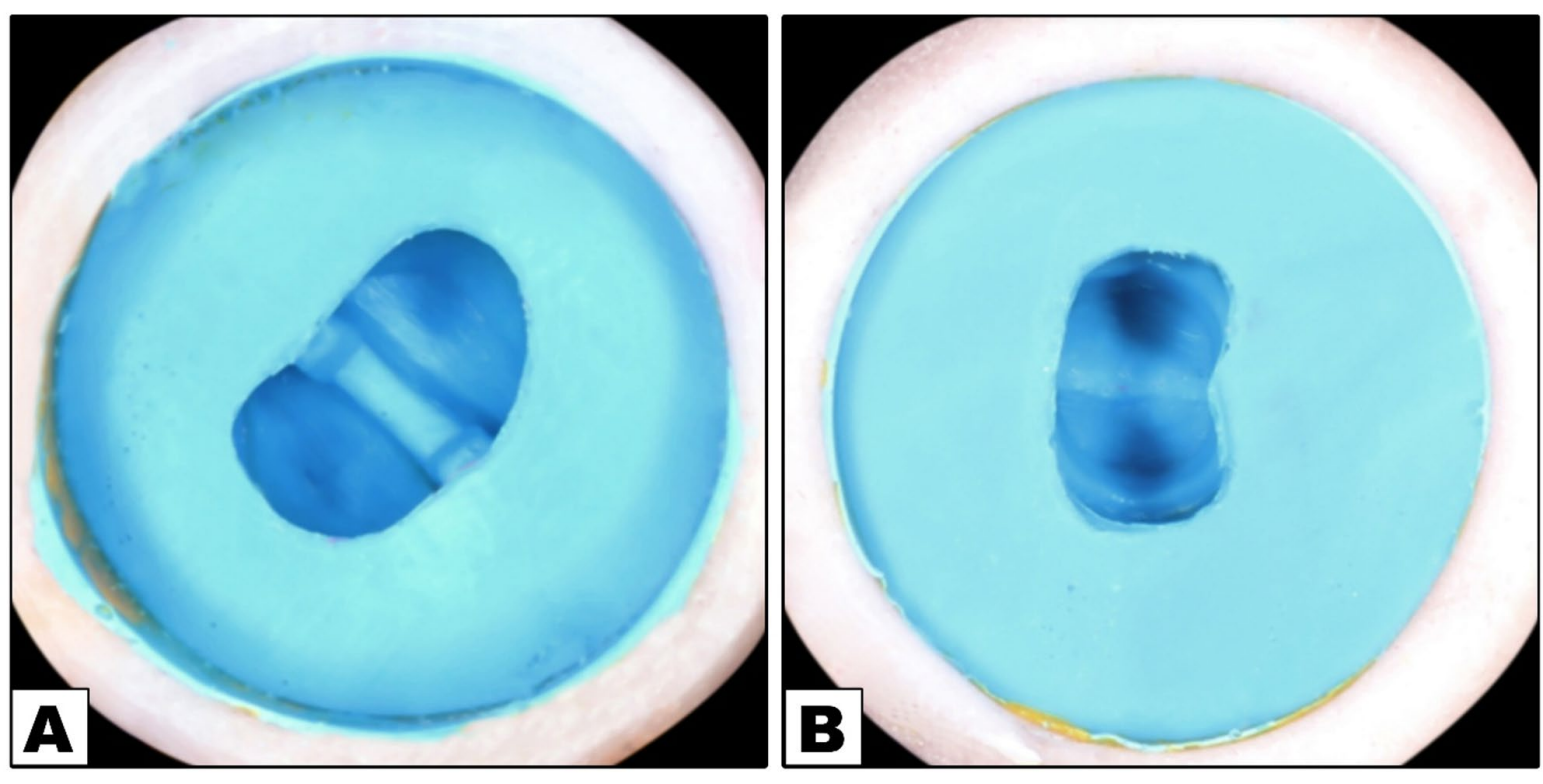
**A** Conventional impression of design 1 and **B** Conventional impression of design 2

Sixteen samples (eight from design 1 and eight from design 2 ) were wrapped by polyethylene sheet that acted as a light-body spacer. Double-mix, two-step 16 silicone putty impressions (HD Elite plus PVS Impression Material normal Set, Germany) were taken individually for 16 samples by custom acrylic trays following the manufacturer’s guidelines [20]. Each impression was poured by Lady Resin color (polyurethane-based resin) according to manufacturer’s instructions under vibration to avoid air inclusion in the mix [21]. The resulting resin models were scanned using a lab desktop scanner (Shinning 3D, AutoScan-DS-EX Pro) to obtain 16 STL files of 16 resin model images (Fig. 2A&B).

#### Digital impression technique

**Fig. 3:**
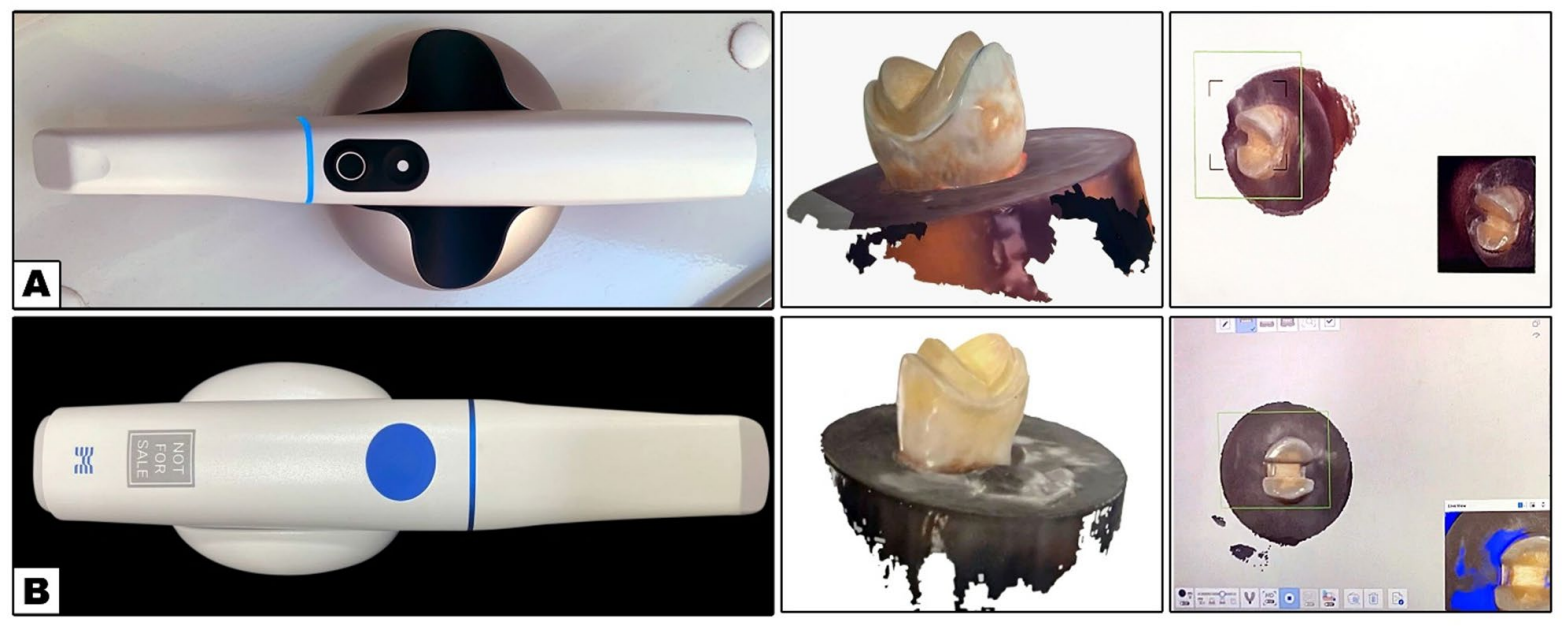
**A** TRIOS 5 (3Shape) scanner and **B** Medit i900 (Medit) scanner

Sixteen intraoral scans of sixteen sample (eight from design 1 and eight from design 2 ) were conducted individually under extraoral conditions utilising a dental intraoral scanner (TRIOS 5, 3Shape, Copenhagen, Denmark**)** (Fig. 3A), Then corresponding exported files (digital virtual models) were used to fabricate the restoration and sixteen intraoral scans of sixteen sample (eight from design 1 and eight from design 2 ) were conducted individually under extraoral conditions utilising a dental intraoral scanner (MEDIT i900, Medit Corp., Seoul, South Korea) (Fig. 3B), then corresponding exported files (digital virtual models) were used to fabricate the restoration.

**Fig. 4:**
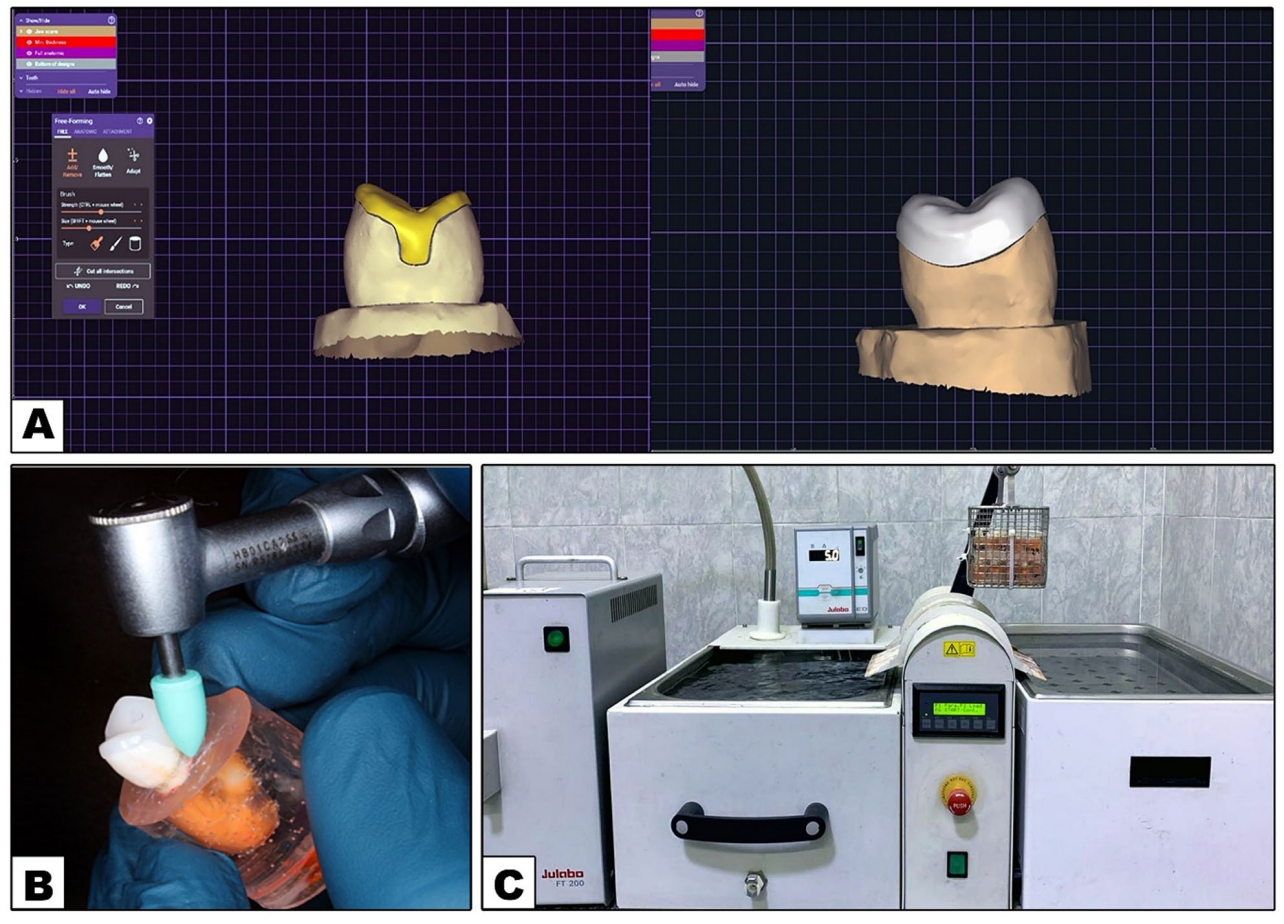
**A** CAD/CAM overlays designing, **B** overlays finishing and polishing after cementation and **C** Thermal cycling process

The CAD/CAM process chain including scanning, designing, and milling processes was followed so each design file was transmitted to the milling machine after it was finished (Fig. 4A). During the design phase using Exocad software (Dental DB 2.2 Valletta Exocad GmbH, Germany), all restorations were standardized with a 50 μm cement space applied to the internal surfaces, while the finish line was maintained at a zero-gap distance (0 μm) without any additional extra cement gap. The milling procedure was started using special burs (CORiTEC milling tools, imes-icore GmbH, Germany). To reduce the possible effect of bur wear on marginal adaptation, the burs were periodically replaced according to the manufacturer’s recommendations throughout the milling procedures. The monolithic blank INDURATE^®^ high-translucent zirconia ( Ø 98.5 × 10 mm) was fixed in the milling machine chamber (CORiTEC 250i, imes-icore, Germany) using a special holder. All milled overlays were then sintered in Zetin ZTCF-30B oven (Zetin Dental, China). The sintering cycle began at room temperature, with a temperature increase of 10 °C per minute until reaching 1500 °C, as per the manufacturer’s specifications. The temperature was held at 1500 °C for two hours, controlled cooling was then performed down to 200 °C at a rate of 5 °C/min, The restorations were subsequently permitted to cool naturally within the furnace to room temperature. The cumulative sintering duration was approximately 9 h, A uniform thin coat of glaze (VITA Akzent Plus, VITA Zahnfabrik, Germany) was added to the surface of each overlay using a clean brush. Once glazing was completed, the restorations were placed on a honey-combed firing tray and transferred to a furnace (Multimat Cube press, Dentsply Sirona, Germany) according to the manufacturer’s guidelines. All milled overlay restorations were checked using a digital caliper to check and confirm the uniform dimensions for each design group.

### Cementation of overlay restorations

Fitting surface of all zirconia overlays were sandblasted by alumina oxide particles (50 μm, 2 bar, 15 s, 10 mm distance) [19], then all restorations were cleaned in an ultrasonic bath using distilled water. Before applying cement, all prepared teeth were cleaned ultrasonically, and then meticulously cleaned with water spray, followed by a gentle drying with oil-free air. For tooth surface treatment, Multilink Primer A and B were applied, equal amounts of each primer (1:1 ratio) were mixed and then applied to the tooth surfaces using a microbrush, scrubbed for 30 s. Excess material was removed with an air stream, and the primer was allowed to self-cure. To facilitate the handling of zirconia restorations, each overlay was grasped by a bond brush with the aid of liqidam. The restoration’s fitting surface was meticulously cleaned with an air-water spray and thereafter dried with a mild air stream. Microbrush was used to apply a thin layer of Monobond N to the pre-treated surface, allow the substance to undergo reaction for 60 s. Subsequently, expel any residual excess using a powerful air stream in accordance with the manufacturer’s guidelines. Upon finishing the surface treatment, a suitable quantity of resin cement (Multilink Speed, Ivoclar Vivadent, Liechtenstein, Switzerland) was injected and uniformly distributed across the fitting surface of the zirconia restoration [22]. Each restoration was meticulously positioned onto its respective prepared tooth, utilising light finger pressure to guarantee accurate initial placement. Subsequently, a specifically engineered loading apparatus was used to deliver axial loading to the cemented restorations under a static load of 5 kg for 10 min [23]. The cement was initially spot cured for 2–3 s at each margin to facilitate the removal of excess cement with a disposable scalpel. Following the removal of excess cement, a light cure was applied for 20–30 s at the edges to accelerate the final setting process. After completing the light-curing steps, the restorations were immediately subjected to finishing and polishing procedures (Fig. 4B). Then specimens were preserved in distilled water at 37 °C for one month and water changed every one week through period of storage. Distilled water was used to maintain standardized hydration conditions and minimize variability associated with different artificial saliva formulations.

### Thermal cycling

The cemented restorations were subjected to thermocycling using a thermal aging device (SD Mechatronic Thermocycler, SD Mechatronic GmbH, Germany) to simulate intraoral conditions. A total of 5000 cycles were applied to constitute around 6 months of clinical service. Thermal cycling was performed between 5 °C and 55 °C with an immersion time of 30 s in each water bath and a transfer time of 10 s [24] (Fig. 4C ).

### Marginal adaptation test

**Fig. 5:**
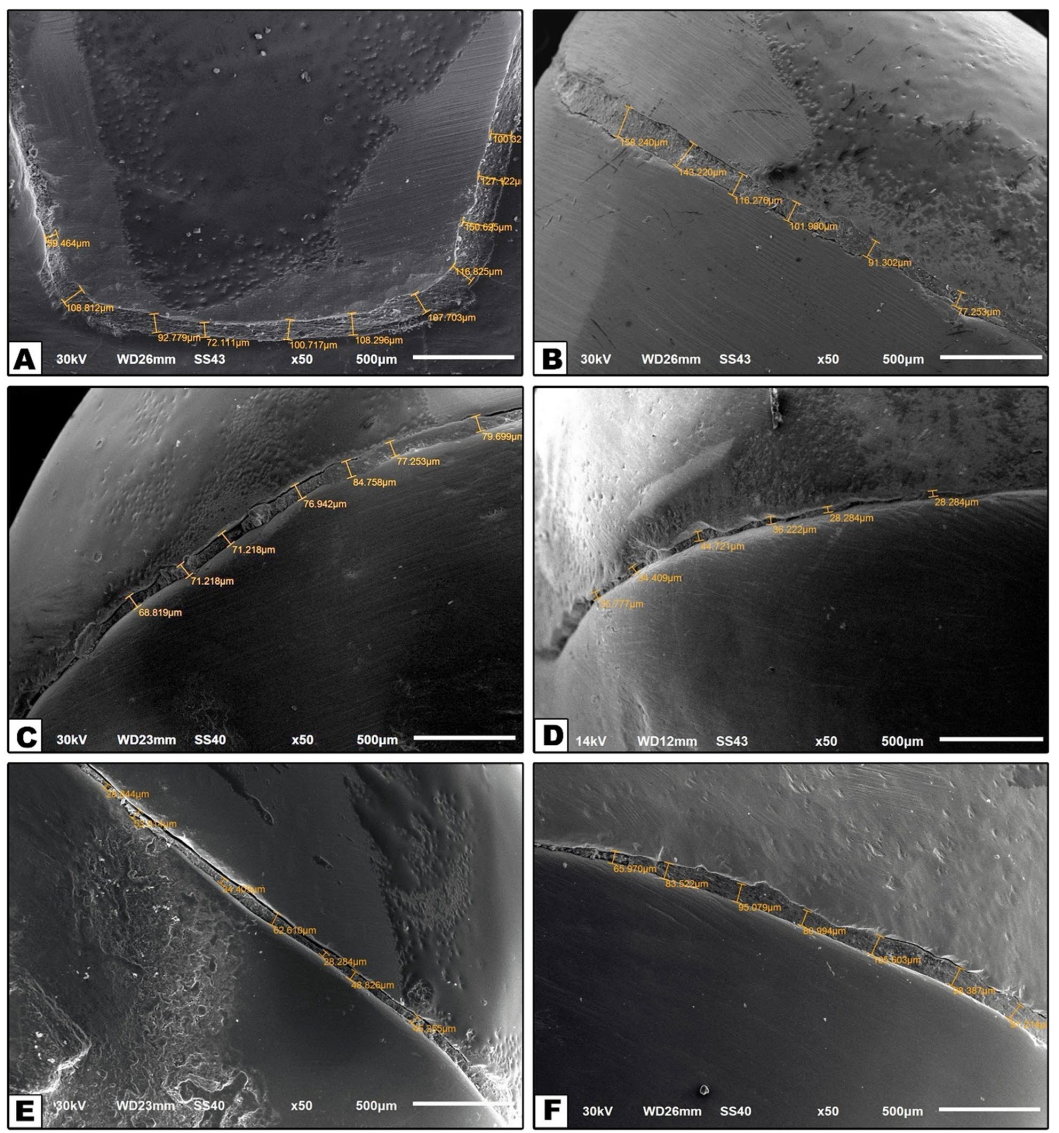
**A**, **B** and **C** Post-aging testing, including marginal adaptation test for design 1 and **D**, **E** and **F** marginal adaptation test for design 2

After cementation and thermocycling, The evaluation of marginal gap was conducted using a Scanning Electron Microscope (SEM) (JEOL JSM-6510LV, JEOL, Tokyo, JAPAN) in Mansoura Microscopy Center, Faculty of Agriculture, Mansoura University. Before SEM analysis, all specimens were sputter-coated with a thin gold layer to augment electrical conductivity. Gold coating was executed utilising a sputter coater (SPI-Module Sputter Coater, SPI Supplies, West Chester, USA). The device operates under vacuum conditions and deposits a uniform 24-karat gold film on the sample surface to ensure high-quality imaging and prevent charging artifacts during scanning electron microscopy. During SEM evaluation, all specimens were secured to an aluminum SEM sample holder using double-sided carbon tape to ensure standardized specimen orientation and positioning throughout image acquisition and measurements. Initially, the image displaying the entire coronal portion of the tooth with restoration was automatically projected onto the computer monitor connected to the microscope. High-resolution SEM images were captured to accurately measure the vertical marginal gap between the prepared tooth margin and the cemented restorations. Each specimen was examined from the buccal, mesial, distal, and palatal aspects. SEM evaluation was performed under standardized operating conditions (30 kV accelerating voltage, working distance 23–26 mm, SEI mode) at ×50 magnification. All measurements were performed directly using the integrated SEM measurement software following calibration according to the microscope scale bar (500 μm) and obtained from multiple predefined points along the tooth-restoration interface using perpendicular measurement orientation to ensure reproducibility and standardization. Each restoration was examined at the four tooth surfaces. For every surface, three predefined points were selected, (e.g., the mesial surface) measurements were recorded at three points : the mesiobuccal, mid-mesial, and mesioplatal points, and five measurements were taken at each point. At each of these points, five successive measurements were recorded to enhance accuracy and reduce measurement variability [24]. Accordingly, a total of 15 measurements per surface and 60 measurements per specimen were obtained. The mean marginal gap value for each surface was calculated from its fifteen readings (Fig. 5).

### Statistical analysis

Data analysis was performed using the Statistical Package for Social Sciences (SPSS) software, version 26 (SPSS Inc., Chicago, IL, USA). The Shapiro–Wilk test was utilized to assess the normality of data distribution. The normally distributed quantitative data were presented as mean ± standard deviation. An independent (unpaired) Student’s t-test was used to compare two independent parametric groups, whereas one-way ANOVA was applied for comparisons among multiple parametric groups exceeding two. A two-way ANOVA was performed to assess the simultaneous effects of many independent variables. Following the identification of a statistically significant difference in the ANOVA analysis, Tukey’s post hoc test was employed for multiple pairwise comparisons. The threshold for statistical significance was established at *p* < 0.05.

## Results

**Fig. 6:**
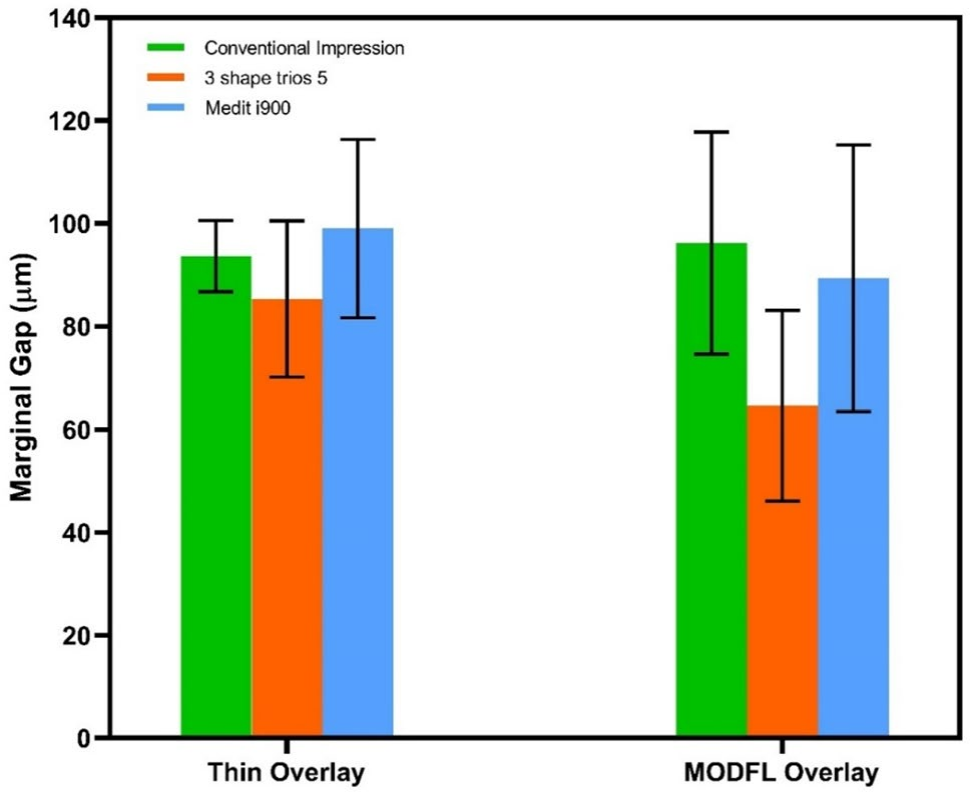
Bar chart showing means of marginal gap values for two partial coverage restorative designs (Thin Overlay and MODFL Overlay) produced using three distinct impression techniques

All mean values fall within clinically acceptable limits. Reduced marginal gap values indicate superior marginal adaptation which is clinically advantageous, in thin overlay design (D1), TRIOS 5 (3Shape) (D1SC1) demonstrated the lowest mean marginal gap (85.34 ± 15.15 μm), indicating the best marginal adaptation among the thin Overlay groups (D1). Conventional impression in D1 (D1C) showed a slightly higher mean value (93.66 ± 6.92 μm) than SC1, while Medit i900 (Medit) (D1SC2) recorded the highest mean marginal gap (99.01 ± 17.36 μm), suggesting comparatively inferior marginal adaptation for D1 Overlays. In MODFL (D2), TRIOS 5 (3Shape) (D2SC1) again showed the lowest marginal gap (64.61 ± 18.51 μm), which is markedly better than the other techniques, Medit i900 (Medit) (SC2) in design 2 (D2SC2) showed an intermediate mean value (89.38 ± 25.89 μm), while Conventional impression(D2C) resulted in the highest marginal gap (96.21 ± 21.57 μm). Overall, TRIOS 5 (3Shape) (SC1) consistently produced the best marginal adaptation, regardless of design type (Table 1; Fig. 6).

**Table 1:** Mean & SD of marginal gap (µm) among the studied groups

Type of Design	Method of taking impression	Mean ± SD
Thin overlay (D1)	Conventional impression (C)	93.66 ± 6.920
	TRIOS 5 (3Shape) (SC1)	85.34 ± 15.15
	Medit i900 (Medit) (SC2)	99.01 ± 17.36
MODFL overlay (D2)	Conventional impression (C)	96.21 ± 21.57
	TRIOS 5 (3Shape) (SC1)	64.61 ± 18.51
	Medit i900 (Medit) (SC2)	89.38 ± 25.89

Independent Sample T-test between D1& D2. Overlay regardless of the method of taking impression revealed that there is no statistical significance difference (*p* = 0.128). The 95% confidence interval of the mean difference ranged from − 2.80 μm to 21.34 μm, crossing zero, which further confirms the absence of a statistically reliable difference between the two design types.

One-way ANOVA performed to assess the effect of Method of taking impression on marginal gap values, regardless the preparation design. The analysis showed a statistically significant difference among the method of taking the impression (*p* = 0.007). This result highlights the Method of taking impression as a key independent factor affecting marginal adaptation, independent of restoration design.

According to Two-way ANOVA, design type showed no statistically significant effect on marginal adaptation (*p* = 0.090), so the difference between two designs alone does not significantly influence marginal adaptation. Method of taking the impression had a statistically significant effect (*p* = 0.005), so the method used to take the impression significantly affects marginal adaptation, confirming that method of taking impression is a key determinant of restoration fit. The interaction effect was not statistically significant (*p* = 0.218), so the method of taking impression especially the influence of scanner type on marginal adaptation is consistent across both designs, meaning that no scanner behaves differently depending on whether the restoration is Thin or MODFL overlay (Table 2).

**Table 2:** Two Way-ANOVA between the type of the design and the method of taking impression

Source	Type III Sum of Squares	df	Mean Square	F	*p*-value
Corrected Model	6212.758	5	1242.552	3.625	0.008
Intercept	371998.935	1	371998.935	1085.205	< 0.001
Design type	1031.844	1	1031.844	3.010	0.090
Method of taking impression	4097.247	2	2048.623	5.976	0.005
Design type * Method of taking impression	1083.667	2	541.833	1.581	0.218
Error	14397.235	42	342.791		
Total	392608.928	48			
Corrected Total	20609.992	47			

The Tukey post-hoc multiple comparison test indicated that statistically significant changes in marginal adaption were mostly related to restorations produced using the SC1, especially in D2. Design 2 overlays generated with SC1(D2SC1) exhibited markedly reduced marginal gap values in comparison to those produced by the conventional impression technique (D2C) (*p* = 0.017) and in relation to D1SC2 (*p* = 0.007), as well as D1 created using the conventional impression method (D1C) (*p* = 0.034). Conversely, the majority of pairwise comparisons between conventional impressions(C) and SC2, irrespective of design type, were not statistically significant, suggesting similar marginal adaptation between the two approaches. No notable variations were seen between D1&D2 overlays while using the same scanner, corroborating the lack of a substantial interaction effect. The Tukey analysis indicated that the enhanced marginal adaption seen in the research is largely due to the use of the SC1, rather than the restoration design, with its benefits becoming more apparent in complex thin conventional overlay restorations (Table 3).

**Table 3:** Tukey post-hoc multiple comparison test

Groups	Groups	Mean Difference	*p*-value	95% Confidence Interval	
				Lower Bound	Upper Bound
D1C	D1SC1	8.32375	0.945	-19.3116	35.9591
	D1SC2	-5.35125	0.992	-32.9866	22.2841
	D2C	-2.54375	1.000	-30.1791	25.0916
	D2SC1	29.04875	0.034	1.4134	56.6841
	D2SC2	4.28625	0.997	-23.3491	31.9216
D1SC1	D1SC2	-13.67500	0.680	-41.3104	13.9604
	D2C	-10.86750	0.847	-38.5029	16.7679
	D2SC1	20.72500	0.242	-6.9104	48.3604
	D2SC2	-4.03750	0.998	-31.6729	23.5979
D1SC2	D2C	2.80750	1.000	-24.8279	30.4429
	D2SC1	34.40000	0.007	6.7646	62.0354
	D2SC2	9.63750	0.901	-17.9979	37.2729
D2C	D2SC1	31.59250	0.017	3.9571	59.2279
	D2SC2	6.83000	0.976	-20.8054	34.4654
D2SC1	D2SC2	-24.76250	0.102	-52.3979	2.8729

## Discussion

Partial coverage indirect restorations are designed to maximize the preservation of natural tooth structure compared to traditional full coverage restorations [19].

Natural teeth of comparable dimensions were selected to possibly mimic clinical processes. This study utilized extracted human premolar teeth due to their bonding properties, modulus of elasticity, and strength, which accurately reflect clinical conditions in contrast to other abutment materials [19, 25].

Each tooth root was integrated within epoxy resin up to 2 mm apical to the cementoenamel junction, with the furcation area entirely hidden to simulate the clinical positioning of the root within the alveolar bone. Epoxy resin was selected for its modulus of elasticity (12 GPa), which closely approximates that of human bone (18 GPa). The preparation was standardized using a dental surveyor [25, 26].

Mishra et al. [20] reported that the putty wash two-step technique utilizing a polyethylene spacer demonstrated superior accuracy compared to the putty wash one step technique across all dimensions. This superiority is attributed to the lack of bulk control in the one step technique, the entrapment of air bubbles, and the pressure areas on the prepared teeth, including the margins, which are replicated with putty rather than syringe materials. Additionally, the simultaneous mixing of putty and syringe materials contributes to the overall distortion of the impression due to the setting distortion of the putty.

Although stone dies exhibited marginally lower gap values compared to polyurethane resin dies, the difference was not statistically significant, suggesting similar performance of both die materials in terms of marginal adaptation. Previous investigations have demonstrated that polyurethane materials may exhibit lower dimensional accuracy and greater dimensional variability compared with Type IV stone. Nevertheless, it has also been reported that dimensional changes in polyurethane can be reduced when the material is reinforced with silica fillers, which may account for the minimal difference observed between the two groups in that study, despite the exact filler content of the polyurethane material used being unknown. Differences between studies may be attributed to variations in scanning and cementation procedures. Polyurethane dies may allow more accurate digital scanning due to their smoother surface characteristics, resulting in improved adaptation of restorations prior to cementation. For this reason, Impressions were poured using Lady Resin, a polyurethane-based resin, to achieve precise and stable dies. Conversely, when restorations are cemented on their corresponding abutments, Type IV stone dies may provide better adaptation. This can be attributed to the minimal setting expansion of Type IV stone (0.09%), which is more advantageous for the seating of indirect CAD/CAM restorations compared to the negligible polymerization shrinkage of polyurethane materials (0.025%) [21].

Digital impression technology is surpassing traditional approaches by facilitating immediate scanning of prepared teeth and minimizing clinical errors. For this purpose CAD-CAM scanner was employed in this study to ensure precision in the technique [25].

Other Findings have shown that intraoral scanners can attain superior accuracy relative to laboratory desktop scanners. This exceptional performance is ascribed to their color streaming technology, which records continuous video with integrated anti-shake stabilization. Furthermore, the scanner generates light at a shorter wavelength, minimizing scattering, refraction, and transmission inaccuracies, thereby improving scanning accuracy [27].

The technology of TRIOS 5 (3Shape) is based on advanced confocal microscopy combined with ultra-fast optical scanning and intelligent image processing. The scanner captures high-resolution 3D data through continuous image acquisition, enabling accurate surface reconstruction with minimal stitching errors. TRIOS 5 (3Shape) is designed to enhance full-arch scanning accuracy, demonstrating high trueness and precision in multiple invitro studies. It demonstrated superior trueness (approximately 54.9 μm) and precision (around 37.8 μm) during full-arch implant scanning [10].

The Medit i900 (Medit) system utilizes triangulation-based structured light scanning technology, combined with high-speed optical image acquisition and continuous video capture to generate detailed three-dimensional digital impressions. The projected structured light pattern is analyzed through triangulation principles to reconstruct accurate surface geometry, while advanced data processing algorithms enhance image stitching and reduce noise. It achieved competitive levels of trueness and precision, ranking second to TRIOS systems as Medit scanners exhibit trueness values of approximately 60.5 μm and precision around 40.6 μm, while noting a tendency toward slight underestimation in full-arch measurements. These findings suggest that although Medit i900 (Medit) provides reliable accuracy for small to moderate scanning spans, minor deviations may occur with extended scanning distances [10].

In the present study INDURATE^®^ high-translucent zirconia was selected due to its enhanced translucency 49%, comparable to lithium disilicate, At the same time, its flexural strength (> 800 MPa) ensures adequate mechanical reliability for clinical applications, including overlays, Additionally The high quality of INDURATE^®^ zirconia is due to the utilization of premium raw materials combined with a highly controlled manufacturing process. The material is produced from 100% Japanese TOSOH yttria-stabilized nano-zirconia powder, which is recognized for its high purity and medical-grade quality. The uniform particle size and homogeneous chemical composition of the zirconia powder contribute to consistent mechanical behavior, marginal adaptation, reduced fracture probability, and enhanced resistance to aging [28].

Lithium disilicate glass ceramic is frequently used for indirect bonded restorations due to its superior mechanical qualities, including strong flexural strength, fracture resistance, and bond strength, which result from its elevated crystalline content (~ 70%). However, fracture of etchable ceramic materials is the primary concern related to posterior teeth, especially when covering one or more cusps or the complete occlusal surfaces. Nonetheless, when meticulously engineered, these materials may offer sustained clinical effectiveness. The constrained flexural strength of materials and resultant fractures require etchable ceramic restorations to be 1.5–2 mm thick. However, Zirconia, the strongest of all current dental ceramics, holds a unique status among metal oxides due to its exceptional mechanical properties, demonstrating a flexural strength of 800–1200 MPa that meets the mechanical load requirements for load-bearing posterior restorations in patients with limited occlusal space. Zirconia, lacking a glassy amorphous phase, cannot be etched or adhesively bonded to the underlying tooth structure as other glass-ceramics can. Consequently, it is regarded as a non-adhesive restorative substance. It has been extensively documented that adherence to the accurate protocol and utilization of adhesive containing the phosphate ester monomer 10-methyacryloyloxydecyl dihydrogen phosphate (10-MDP) enhances zirconia crown cementation. Furthermore, adhering zirconia to the dental substrate, following a specific bonding protocol, establishes a strong bond to the tooth structure as a partial or full coverage restoration [17, 19].

Shokry et al. [22] noted that monolithic zirconia was selected for its superior flexural strength and fracture toughness, and it can be employed in thicknesses as minimal as 0.5 mm. In contrast to glass ceramic, zirconia is impervious to etching due to the absence of a glassy matrix, hence eliminating the possibility of employing conventional adhesive techniques; consequently, a self-adhesive resin cement (Multilink Speed) was chosen for this study. This cement was selected for its beneficial physical characteristics and simplicity of application. Resin cements with the MDP monomer can achieve superior adhesion to zirconia due to the interaction between the hydroxyl groups on the zirconia surface and the phosphate ester group of the MDP molecules. The acidic monomers of the self-adhesive resin cement can effectively wet and adhere to the exposed hydrophilic inorganic fillers generated by air abrasion of the zirconia surface. The Multilink Speed exhibited the highest retention strength value and This finding was supported by previous research employing MDP-containing primers on air-abraded zirconia surfaces to improve the physicochemical interaction between zirconia and resin cement. The MDP molecule possesses a functional phosphoric acid group, while the ceramic primer (Monobond N) comprises methacrylate monomers, collectively establishing a stable bond that resists hydrolysis and guarantees persistent adhesion to zirconium oxide.

The materials are affected by temperature variations in the mouth cavity. This study involved thermocycling for 5000 cycles at bath temperatures of 5 °C and 55 °C, with a dwell time of 20 s, to simulate the temperature fluctuations of the oral cavity over six months of clinical service [24].

The definition of misfit varies among researchers and has not been standardized. Various studies have evaluated fit by characteristics including marginal adaptation, internal adaptation, vertical seating, radiographic appearance, and clinical suitability, resulting in inconsistencies, particularly when comparing findings across study. Reported values of marginal gaps also show wide variation, ranging from 35.4 to 246 μm. Currently, no universal consensus exists regarding a clinically acceptable marginal discrepancy; however, a recent study indicated that overall mean marginal fit values remained below 120 μm. This variability can be attributed to multiple influencing factors, including differences in restoration fabrication methods, tooth preparation designs, measurement locations, and measurement techniques [29, 30].

The chosen method for assessing marginal gap was direct evaluation with a Scanning electron microscope (SEM), which facilitated standardized measurements by placing the reconstructed teeth on a base. This technique is limited by the challenge of differentiating between the tooth structure and the lowest portion of the finish line margin, as well as its two-dimensional evaluative method, which relies on measurements from specific points and may not adequately capture three-dimensional internal variations. In contrast, contemporary techniques like Micro-CT offer non-destructive three-dimensional volumetric measurement, facilitating a more comprehensive evaluation of both marginal and interior adaptation. Conversely, it offers numerous advantages, including rapid technique and cost-effectiveness, as it does not necessitate supplementary operations like specimen sectioning. The risk of cumulative errors is reduced compared to other strategies involving multi-step procedures. Furthermore, a single operator conducted all measurements while being blinded to the restoration materials. The measurements of marginal gaps were determined to be conducted post-cementation of overlay restorations to replicate clinical settings. A cement gap of 50 μm was created to establish a uniform layer of cement, which prior research has validated to possess marginal fit values within clinically acceptable limits. As meeting marginal conditions post-cementation is a critical determinant for the long-term clinical effectiveness of permanent restorations, Marginal gaps are influenced by various aspects, including the design of preparation, fabrication techniques, measurement methods, and the materials employed [24, 31].

The null hypothesis of this study was partially accepted; as no significant differences in marginal gap were found among the two preparation designs, and no significant interaction effect between design type and impression method was detected, while a significant effect was detected for the method of impression taking, confirming its influence on marginal gap values.

The design of the preparation is essential for assessing the marginal adaption of indirect restorations. Marginal adaptation is a crucial factor influencing the long-term prognosis and clinical outcomes of ceramic restorations [2].

In this study, an independent samples t-test conducted to evaluate whether the type of restoration design (Thin Overlay versus MODFL Overlay), has a significant effect on marginal gap values irrespective of method of taking impression used. The analysis revealed that there is no statistical significance (*p* = 0.128) between 2 designs. The 95% confidence interval of the mean difference ranged from − 2.80 μm to 21.34 μm, crossing zero, which further confirms the absence of a statistically reliable difference between the two design types. This finding was further validated by two-way ANOVA which confirmed that design type showed no statistically significant effect on marginal adaptation (*p* = 0.090), so the difference between D1, D2 alone does not significantly influence marginal adaptation.

Additionally, Post hoc analysis using Tukey’s test revealed that no statistically significant differences were observed in marginal adaptation between the thin overlay (D1) and MOD-FL (D2) across the different scanning and impression techniques. Specifically, for the TRIOS 5 (3Shape) (*P* = 0.242), Medit i900 (Medit) (*P* = 0.901), and conventional impressions (*P* = 1.000), the measured marginal gaps were comparable, indicating that the design type did not influence the marginal adaptation of the restorations.

The analysis of the present study disagrees with other study that showed that the circumferential shoulder design exhibited superior marginal adaptation compared to the conventional overlay. This superiority is attributed to the circumferential shoulder’s straightforward preparation characteristics, which encompass a flat, smooth occlusal reduction, absence of retentive features, and minimal internal angles. These characteristics enhance digital workflow processes, including rapid scanning during digital impression capture, efficient software design, and enabling milling burs to replicate the details of overlays, hence leading to a diminished marginal gap value [32].

Other study showed that after cementation, the circumferential shoulder design may allow for a better cement flow compared to other designs. This property is lacking for thin conventional design leading to an increase in hydraulic pressure and discharge of excess cement, thus the marginal gap value of their overlay restorations is increased after self-adhesive resin cementation [33]. Moreover, the conventional design includes inter axial tooth structure reduction and formation of occlusal isthmuses leading to increasing the friction during restoration insertion and thus has a negative impact on the marginal fit [2]. These results disagree with the result of the present study as no significant differences in marginal gap were found among the two preparation designs.

Falahchai et al. [34] stated that the most complex preparation design for overlay restorations yields the lowest marginal adaptation behavior and in comparison of the marginal gap between the conservative overlay and traditional preparation, the conservative preparation exhibited a significantly superior marginal adaptation both before and after cementation, but in the present study design type showed no statistically significant effect on marginal adaptation, TRIOS 5 (3Shape) produced the best marginal adaptation, regardless of design type and the interaction effect was not statistically significant (*p* = 0.218), so the method of taking impression especially the influence of scanner type on marginal adaptation is consistent across both designs, meaning that no scanner behaves differently depending on whether the restoration is Thin or MODFL overlay, no notable variations were seen between Thin and MODFL overlays while using the same scanner, corroborating the lack of a substantial interaction effect.

According to one-way ANOVA there is statistically significant difference among the impression techniques (*p* = 0.007). This result highlights the method of taking impression as a key independent factor affecting marginal adaptation, independent of restoration design. Additionally, a two-way ANOVA confirmed this significance as method of taking impression had a statistically significant effect (*p* = 0.005), so the null hypnosis was rejected, as the method used to take the impression significantly affects marginal adaptation, confirming that method of taking impression is a key determinant of restoration adaptation.

In Thin Overlay design(D1), TRIOS 5 (3Shape) (D1SC1) demonstrated the lowest mean marginal gap (85.34 ± 15.15 μm), indicating the best marginal adaptation among the Thin Overlay groups and Conventional impression in D1(D1C) showed a slightly higher mean value (93.66 ± 6.92 μm) than SC1. In MODFL Overlay design(D2), TRIOS 5 (3Shape) (D2SC1) again showed the lowest marginal gap (64.61 ± 18.51 μm), which is markedly better than the other techniques while Medit i900 (Medit) (D2SC2) showed an intermediate mean value (89.38 ± 25.89 μm) and Conventional impression(D2C) resulted in the highest marginal gap (96.21 ± 21.57 μm). According to Tukey post-hoc multiple comparison test, MODFL overlays generated with TRIOS 5 (3Shape) (D2SC1) exhibited markedly reduced marginal gap values in comparison to those produced by the conventional impression technique(D2C) (*p* = 0.017), as well as Thin overlays created using the conventional impression method (D1C)(*p* = 0.034) and these results were in accordance with other studies which reported that digital techniques achieved superior marginal adaptation than conventional impression [29].

When comparing the impression techniques for the Thin Overlay design, the conventional impression (D1C) showed lower mean marginal gap (93.66 ± 6.92 μm) than Medit i900 (Medit) (D1SC2) which recorded the highest mean value (99.01 ± 17.36 μm). However, no statistically significant differences were observed between two groups, as confirmed by Tukey’s post-hoc multiple comparison test (*p* = 0.992). the majority of pairwise comparisons between conventional impressions (C) and the Medit i900 (Medit) (SC2), irrespective of design type, were not statistically significant, suggesting similar marginal adaptation between the two approaches. These findings align with other study which indicated that neither digital intraoral impressions nor CAD/CAM systems, irrespective of the scanner model employed, demonstrated enhanced accuracy relative to conventional impression methods concerning the marginal adaptation of ceramic inlay-onlays [29].

In Thin Overlay design, TRIOS 5 (3Shape) (D1SC1) demonstrated the lowest mean marginal gap (85.34 ± 15.15 μm), indicating the best marginal adaptation among the Thin Overlay groups. For Thin Overlay restorations, digital scanning—particularly TRIOS 5 (3Shape)—appears to provide superior marginal adaptation compared to Medit i900 (Medit) which showed higher variability and mean gap values (99.01 ± 17.36), these results suggested that scanner accuracy plays a critical role when dealing with more complex cavity designs, but there is no statistical difference. For MODFL overlays, the advantage of TRIOS 5 (3Shape) is even more pronounced. Overall, TRIOS 5 (3Shape) produced the best marginal adaptation, regardless of design type. The Tukey post-hoc multiple comparison test indicated that statistically significant changes in marginal adaption were mostly related to restorations produced using the TRIOS 5 (3Shape) scanner, especially in MODFL overlay design. MODFL overlays generated with TRIOS 5 (3Shape) (D2SC1) exhibited markedly reduced marginal gap values in comparison to thin overlays scanned with Medit i900 (Medit) (D1SC2) as confirmed by Tukey’s post-hoc multiple comparison test (*p* = 0.007) and These findings are consistent with Ahmed et al. [11] who reported superior trueness and precision for TRIOS 5 (3Shape) compared to Medit i900 (Medit), confirming its higher accuracy and performance .

These findings also are consistent with extensive literature identifying TRIOS scanners as the most accurate intraoral scanning systems. In controlled invitro experiments, TRIOS 5 (3Shape) has repeatedly shown high levels of precision and accuracy, as reported by Jain et al. [10] These findings also are consistent with Vasilescu et al. [12], who reported that TRIOS 5 (3Shape) showed the smallest mean deviation (~ 112 μm) indicating superior accuracy among the scanners tested.

## Limitations

1. It was nearly impossible to obtain ideal uniformity in the natural teeth, the used teeth had a close range of Dimensions.
2. Limited aging 5000 cycles was simulated; only 6 months of function was applied.
3. The study did not consider the potential differences between intraoral and extraoral conditions; therefore, the impact of oral environmental factors on the accuracy of impression techniques and the marginal integrity of partial-coverage ceramic restorations could not be assessed. In addition, specimens were stored in distilled water under standardized conditions rather than artificial saliva, which may not completely simulate the clinical oral environment.
4. Only two intraoral scanners were used in this study.

## Conclusions

1. Digital impressions improved marginal integrity of overlay restorations and offer a clinically effective alternative to conventional silicone impressions within a digital workflow.
2. The method used to take the impression significantly affects marginal adaptation, confirming that method of taking impression is a key determinant of restoration fit but Design type showed no statistically significant effect on marginal adaptation.
3. The method of taking impression especially the impact of scanner type on marginal adaptation is consistent across both designs, meaning that no scanner behaves differently depending on whether the restoration is Thin conventional or MODFL overlay.
4. The TRIOS 5 (3 Shape) scanner demonstrated improved marginal adaptation across both preparation designs compared to the other techniques. This may be attributed to its high trueness and precision, as well as its confocal microscopy-based image acquisition technology, with the effect becoming more evident in complex designs.
5. All mean values of marginal gap fall within clinically acceptable limits.

## Data Availability

The datasets generated and/or analyzed during the current study are available from the corresponding author “Ahmed Adly” upon request.

## Abbreviations

IOS: Intraoral scanner
CAD/CAM: Computer-aided design/ Computer-aided manufacturing
Vps: Vinyl Poly siloxane impression material
SEM: Scanning Electronic Microscopy
CEJ: Cemento-enamel junction
PDL: periodontal ligament
Um: Micron (s)
ANOVA: Analysis of Variance
SD: Standard deviation
